# Characterization and Modelling of Manufacturing–Microstructure–Property–Mechanism Relationship for Advanced and Emerging Materials

**DOI:** 10.3390/ma16072737

**Published:** 2023-03-29

**Authors:** Lihong Su, Peitang Wei, Xing Zhao, Hui Wang

**Affiliations:** 1 School of Mechanical, Materials, Mechatronic and Biomedical Engineering, University of Wollongong, Wollongong, NSW 2522, Australia; 2 State Key Laboratory of Mechanical Transmissions, Chongqing University, Chongqing 400044, China; 3 State Key Laboratory of High Performance Complex Manufacturing, Central South University, Changsha 410083, China; 4 Institute of Industrial Science (IIS), The University of Tokyo, Kashiwa 277-8574, Chiba, Japan

Depending on the state of its raw materials, final products, and processes, materials manufacturing can be classified into either top-down manufacturing and bottom-up manufacturing, or subtractive manufacturing (SM) and additive manufacturing (AM). Some important top-down manufacturing methods include casting [1], welding [2], hot rolling [3], cold rolling [4], cryo-forming [5], heat treatment [6], equal channel angular pressing [7], high pressure torsion, [8], and accumulative roll bonding [9], while some important bottom-up manufacturing techniques include powder metallurgy sintering [10,11,12] and filtered arc deposition [13]. The disadvantages of bottom-up manufacturing, in comparison to top-down manufacturing, are its limitations in fabricating large samples and its inability to avoid contamination and residual porosity in its final products. Recently, the AM approach has attracted increasing interest, and some important AM processes include wire-arc-based methods [14,15], laser-based AM methods [16], electron-beam-based AM methods [17], and cold spraying [18]. Regarding the development of the materials, both conventional materials with novel structures (such as nanograined microstructures or ultrafine-grained microstructures [19,20,21,22]) and novel materials (such as high entropy alloys with more than four principal alloying elements [23]) have become popular for material scientists and engineers, owing to their attractive physical and mechanical properties. In addition to experimental characterizations, advanced modelling also plays a critical role, particularly in revealing the phenomena that are hardly observed in experiments. Some important modelling tools that are related to this manufacturing include the finite element method (FEM) [24], crystal plasticity FEM [25], discrete element method (DEM) [26], molecular dynamic (MD) model [27], and atomistic-continuum coupled multiscale model [28]. With the quick development of computational techniques, these models are providing increasing contributions to our understanding of the manufacturing–structure–property–mechanism relationships between various materials under different conditions. This Special Issue has collected the recent advances and investigations within the research that has been filed on manufacturing, covering all of these above mentioned topics.

Hedhibi et al. [29] studied the influence of pseudo-ternary oxides on mechanical properties and microstructures, by comparing the activating tungsten inert gas (ATIG) weld with the conventional tungsten inert gas (TIG) weld. They optimized the composition of the flux and found an improvement in the ultimate tensile strength (UTS), from about 571 MPa for the conventional TIG weld to about 600 MPa for the optimal ATIG weld. Ahmed et al. [30] developed a novel refilling technique for the friction stir spot welding (FSSW) joints of AA6082-T6 sheets. The mechanical testing showed higher-bearing tensile shear loads in all the refilled FSSW joints than those that were given by the as-received FSSW joints. In order to improve the mechanical and tribological performances of an AZ91 Mg alloy, Ataya et al. [31] added short carbon fibers to the AZ91 matrix. They observed a large influence of the carbon fibers’ orientation on both the compressive strength and the wear resistance of the Mg composite; however, they did not observe an obvious difference in the hardness. The paper published by Wan et al. [32] is about a laser-based AM of an Mg alloy. They successfully improved the wear and corrosion resistance of an AZ91D alloy by introducing an Al-Si alloy coating with an addition of Y_2_O_3_, using a laser cladding process. Yin et al. [33] studied the microstructures and textures of various automobile steels and their influences on quasistatic tensile deformation behavior, with a strain rate of 0.001 s^−1^ and dynamic tensile deformation behaviors with a wide strain rate range, varying from 33 to 600 s^−1^. Zhang et al. [34] investigated the relationship between an ultrasonic vibration treatment and the microstructure evolution during the high-temperature forming process of 9310 steel. It was observed that the flow stress of the 9310 steel decreased with an increase in the deformation temperature or a decrease in the strain rate. Kubiak and Lesnikowski [35] investigated the influence of mechanical deformation on the characteristic impedance of sewed textile signal lines (TSLs). Regardless of the tensile forces, only the substrate weave was found not to affect the characteristic impedance change.

Chen et al. [36] studied the vibration characteristics of submarine-like structures with laminated materials that consisted of spherical, cylindrical, and cone shells with multiple built-in annular plates, based on a numerical model. Li et al. [37] developed a numerical discrete model that was based on a meshless Chebyshev-RPIM shape function, in order to study the vibration of a rotating cross-ply laminated combined conical–cylindrical shell in a thermal environment. In their study, Zhang et al. [38] focused on the vibration characteristics of a laminated composite double cylindrical shell system (LCDCSS), coupled with a variable number of annular plates. Dzwierzynska and Lechwar [39] proposed an algorithmic-aided approach for the design and optimization of the curvilinear steel bar structures of unit roofs, and their structural analysis was further verified by an FEM analysis, taking both the permanent and environmental loads into account. With the help of a computational fluid dynamics (CFD) model, Li et al. [40] simulated a high-pressure hydrogen flow through their newly proposed Tesla-type depressurization structure. They found that this pressure could be reduced by 237% if the standard orifice plate was replaced with a Tesla-type orifice structure. Furthermore, the subject of surface roughness in materials and manufacturing engineering has attracted increasing attention in recent years [41]. Lu et al. [42] studied the surface roughness of face gears, based on a non-contact measurement that was obtained via 3D optical scanning and FEM simulations. The paper by Yang et al. [43] is about the rough surface characterization parameter set (CPS) and redundant parameter set (RPS) that are used for surface modeling and performance. They successfully proposed a model for a performance evaluation of different workpiece surfaces, based on their capacity to fully cover the surface topography information. Li et al. [44] conducted MD simulations in order to study the hydrogen-induced dislocation nucleation (DN) and plastic deformation of <001> and <1–10> grain boundaries (GBs) in nickel bicrystals. Additionally, they also studied the influence of grain size on the hydrogen embrittlement (HE) of nanograined iron materials, which was also based on MD simulations [45]. Their study indicated that grain refinement could be an effective strategy for resisting H-induced brittle failure, owing to the fact that finer materials have a lower H concentration at the GBs, and an improved GB-mediated intergranular deformation, thus resulting in a lesser possibility of initiating cracks.

The variety and quality of all these papers are addressed to both academic and industrial researchers who are looking for new information that can contribute to the advancement of future research in these highly challenging fields. It is our hope, as guest editors, that you find this volume interesting. We would like to express our sincere gratitude to the authors for their contributions and cooperation during the editorial process. We are indebted to the reviewers for their constructive suggestions and comments. We thank the editorial team for their strong support throughout the entire process.

## References

[1] Su L., Li H., Lu C., Li J., Fletcher L., Simpson I., Barbaro F., Zheng L., Bai M., Shen J. (2016). Transverse and z-Direction CVN Impact Tests of X65 Line Pipe Steels of Two Centerline Segregation Ratings. Metall. Mater. Trans. A.

[2] Su L., Fei Z., Davis B., Li H., Bornstein H. (2021). Digital Image Correlation Study on Tensile Properties of High Strength Quenched and Temped Steel Weld Joints Prepared by K-TIG and GMAW. Mater. Sci. Eng. A.

[3] Deng G.Y., Tieu A.K., Su L.H., Zhu H.T., Reid M., Zhu Q., Kong C. (2019). Microstructural study and residual stress measurement of a hot rolling work roll material during isothermal oxidation. Int. J. Adv. Manuf. Technol..

[4] Deng G.Y., Tieu A.K., Si L.Y., Su L.H., Lu C., Wang H., Liu M., Zhu H.T., Liu X.H. (2014). Influence of cold rolling reduction on the deformation behaviour and crystallographic orientation development. Comput. Mater. Sci..

[5] Xiong H., Su L., Kong C., Yu H. (2021). Development of High Performance of Al Alloys via Cryo-Forming: A Review. Adv. Eng. Mater..

[6] Su L., Lu C., Tieu K., Deng G. (2013). Annealing Behavior of Accumulative Roll Bonding Processed Aluminum Composites. Steel Res. Int..

[7] Deng G., Lu C., Su L., Tieu A.K., Li J., Liu M., Zhu H.T., Liu X.H. (2014). Influence of outer corner angle (OCA) on the plastic deformation and texture evolution in equal channel angular pressing. Comput. Mater. Sci..

[8] Deng G., Bhattacharjee T., Chong Y., Zheng R., Bai Y., Shibata A., Tsuji N. (2020). Influence of Fe addition in CP titanium on phase transformation, microstructure and mechanical properties during high pressure torsion. J. Alloy. Compd..

[9] Su L., Lu C., Deng G., Tieu K., Sun X. (2013). Microstructure and Mechanical Properties of 1050/6061 Laminated Composite Processed by Accumulative Roll Bonding. Rev. Adv. Mater. Sci..

[10] Vo T.D., Tieu A.K., Wexler D., Su L., Nguyen C., Deng G. (2022). Fabrication and Characterization of a Low-Cost Co-Free Al0.8CrFeNi2.2 Eutectic High Entropy Alloy Based Solid Self-Lubricating Composite; Microstructure, Mechanical and Wear Properties. J. Alloys Compd..

[11] Deng G., Tieu A.K., Su L., Wang P., Wang L., Lan X., Cui S., Zhu H. (2020). Investigation into reciprocating dry sliding friction and wear properties of bulk CoCrFeNiMo high entropy alloys fabricated by spark plasma sintering and subsequent cold rolling processes: Role of Mo element concentration. Wear.

[12] Deng G., Tieu A.K., Lan X., Su L., Wang L., Zhu Q., Zhu H. (2020). Effects of normal load and velocity on the dry sliding tribological behaviour of CoCrFeNiMo0.2 high entropy alloy. Tribol. Int..

[13] Sun Y., Lu C., Yu H., Tieu A.K., Su L., Zhao Y., Zhu H., Kong C. (2015). Nanomechanical properties of TiCN and TiCN/Ti coatings on Ti prepared by Filtered Arc Deposition. Mater. Sci. Eng. A.

[14] Wang J., Pan Z., Wang L., Su L., Carpenter K., Wang J., Wang R., Li H. (2020). In-Situ Dual Wire Arc Additive Manufacturing of NiTi-Coating on Ti6Al4V Alloys: Microstructure Characterization and Mechanical Properties. Surf. Coat. Technol..

[15] Wang J., Pan Z., Wang Y., Wang L., Su L., Cuiuri D., Zhao Y., Li H. (2020). Evolution of crystallographic orientation, precipitation, phase transformation and mechanical properties realized by enhancing deposition current for dual-wire arc additive manufactured Ni-rich NiTi alloy. Addit. Manuf..

[16] Yang Y., Hu J., Liu X.Y., Xu W., Li B., Ling G.P., Pang X.Y., Tian Y.Z. (2022). Post treatment of an additively manufactured composite consisting of 304L stainless steel and CoCrFeMnNi high-entropy alloy. Mater. Sci. Eng. A.

[17] Zhao X., Li S., Zhang M., Liu Y., Sercombe T.B., Wang S., Hao Y., Yang R., Murr L.E. (2016). Comparison of the microstructures and mechanical properties of Ti–6Al–4V fabricated by selective laser melting and electron beam melting. Mater. Des..

[18] Gao P., Zhang C., Wang R., Deng G., Li J., Su L. (2023). Tamping effect during additive manufacturing of copper coating by cold spray: A comprehensive molecular dynamics study. Addit. Manuf..

[19] Su L., Lu C., Deng G., Tieu K. (2014). Microstructure and Mechanical Properties of AA5005/AA6061 Laminated Composite Processed by Accumulative Roll Bonding. Metall. Mater. Trans. B.

[20] Su L.H., Lu C., Tieu A.K., He L.Z., Zhang Y., Wexler D. (2011). Vacancy-assisted hardening in nanostructured metals. Mater. Lett..

[21] Deng G., Chong Y., Su L., Zhan L., Wei P., Zhao X., Zhang L., Tian Y., Zhu H., Tsuji N. (2022). Mechanisms of remarkable wear reduction and evolutions of subsurface microstructure and nano-mechanical properties during dry sliding of nano-grained Ti6Al4V alloy: A comparative study. Tribol. Int..

[22] Su L.H., Lu C., Deng G.Y., Tieu K., Zhang L.C., Guagliardo P., Samarin S.N., Williams J.F. (2014). Vacancy-Type Defects Study on Ultra-Fine Grained Aluminum Processed by Severe Plastic Deformation. Sci. Adv. Mater..

[23] Tian Y.-Z., Peng S.-Y., Chen S.-F., Gu Z.-J., Yang Y., Shang X.-L., Deng G.-Y., Su L.-H., Sun S.-J. (2022). Temperature-dependent tensile properties of ultrafine-grained C-doped CoCrFeMnNi high-entropy alloy. Rare Met..

[24] Deng G.Y., Zhu H.T., Tieu A.K., Zhu Q., Su L.H., Reid M., Wei P.T., Zhang L., Wang H., Zhang J. (2017). Numerical Evaluation of a High Speed Steel Work Roll during Hot Strip Rolling Process. Mater. Sci. Forum.

[25] Deng G.Y., Lu C., Tieu A.K., Su L.H., Huynh N.N., Liu X.H. (2010). Crystal plasticity investigation of friction effect on texture evolution of Al single crystal during ECAP. J. Mater. Sci..

[26] Nguyen V.D.X., Tieu A.K., Andre D., Su L., Zhu H. (2021). Discrete Element Method using Cohesive Platic Beam for Modeling Elasto-Plastic Deformation of Ductile Materials. Comput. Part. Mech..

[27] Zheng X., Su L., Deng G., Zhang J., Zhu H., Tieu A.K. (2021). Study on Lubrication Characteristics of C4-Alkane and Nanoparticle during Boundary Friction by Molecular Dynamics Simulation. Metals.

[28] Zhang J., Zhang L., Tieu A.K., Michal G., Zhu H.T., Deng G.Y. (2016). Finite-Temperature Multiscale Simulations for 3D Nanoscale Contacts. Appl. Mech. Mater..

[29] Hedhibi A.C., Touileb K., Djoudjou R., Ouis A., Alrobei H., Ahmed M.M.Z. (2021). Mechanical Properties and Microstructure of TIG and ATIG Welded 316L Austenitic Stainless Steel with Multi-Components Flux Optimization Using Mixing Design Method and Particle Swarm Optimization (PSO). Materials.

[30] Ahmed M.M.Z., Seleman M.M.E.-S., Ahmed E., Reyad H.A., Alsaleh N.A., Albaijan I. (2022). A Novel Friction Stir Deposition Technique to Refill Keyhole of Friction Stir Spot Welded AA6082-T6 Dissimilar Joints of Different Sheet Thicknesses. Materials.

[31] Ataya S., Seleman M.M.E.-S., Latief F.H., Ahmed M.M.Z., Hajlaoui K., Soliman A.M., Alsaleh N.A., Habba M.I.A. (2022). Wear Characteristics of Mg Alloy AZ91 Reinforced with Oriented Short Carbon Fibers. Materials.

[32] Wan X., Tian C., Li Y., Zhou J., Qian S., Su L., Wang L. (2023). Effect of Y_2_O_3_ Addition on Microstructure and Properties of Laser Cladded Al-Si Coatings on AZ91D Magnesium Alloy. Materials.

[33] Yin S., Xue Y., Cui H., Pei X., Hu C., Wang Y., Tian Q. (2022). Effect of Material Anisotropy on the Mechanical Response of Automotive Steel under High Strain Rates. Materials.

[34] Zhang Y., Zhou W., Tang J., He Y. (2022). Understanding Effects of Ultrasonic Vibration on Microstructure Evolution in Hot Forming Process via Cellular Automata Method. Materials.

[35] Kubiak P., Leśnikowski J. (2022). Influence of Mechanical Deformations on the Characteristic Impedance of Sewed Textile Signal Lines. Materials.

[36] Chen Z., Zhong R., Hu S., Qin B., Zhao X. (2022). Effect of Multiple Annular Plates on Vibration Characteristics of Laminated Submarine-Like Structures. Materials.

[37] Li Z., Hu S., Zhong R., Qin B., Zhao X. (2022). Meshless Chebyshev RPIM Solution for Free Vibration of Rotating Cross-Ply Laminated Combined Cylindrical-Conical Shells in Thermal Environment. Materials.

[38] Zhang Y., Shi D., He D. (2022). Vibration Characteristics of a Laminated Composite Double-Cylindrical Shell System Coupled with a Variable Number of Annular Plates. Materials.

[39] Dzwierzynska J., Lechwar P. (2022). Algorithmic-Aided Approach for the Design and Evaluation of Curvilinear Steel Bar Structures of Unit Roofs. Materials.

[40] Li B., Liu Y., Li J., Liu B., Wang X., Deng G. (2022). Investigation of a Novel Hydrogen Depressurization Structure Constituted by an Orifice Plate with Tesla-Type Channels. Materials.

[41] Nie N., Su L., Deng G., Li H., Yu H., Tieu A.K. (2021). A review on plastic deformation induced surface/interface roughening of sheet metallic materials. J. Mater. Res. Technol..

[42] Lu X., Zhao X., Hu B., Zhou Y., Cao Z., Tang J. (2022). A Measurement Solution of Face Gears with 3D Optical Scanning. Materials.

[43] Yang D., Tang J., Xia F., Zhou W. (2022). Rough Surface Characterization Parameter Set and Redundant Parameter Set for Surface Modeling and Performance Research. Materials.

[44] Li J., Wu Z., Teng L., Deng G., Wang R., Lu C., Li W., Huang X., Liu Y. (2022). Hydrogen-Induced Dislocation Nucleation and Plastic Deformation of <001> and <1–10> Grain Boundaries in Nickel. Materials.

[45] Li J., Wu Z., Wang F., Zhang L., Zhou C., Lu C., Teng L., Lin Q. (2022). Study on the Hydrogen Embrittlement of Nanograined Materials with Different Grain Sizes by Atomistic Simulation. Materials.

